# Brief repeated virtual nature contact for three weeks boosts university students' nature connectedness and psychological and physiological health during the COVID-19 pandemic: A pilot study

**DOI:** 10.3389/fpubh.2022.1057020

**Published:** 2023-01-12

**Authors:** Sam S. S. Lau, Sharron S. K. Leung, Jonathan W. C. Wong, Terence C. P. Lee, Stephen R. Cartwright, Janet T. C. Wong, Jackie Man, Ethan Cheung, Regene P. W. Choi

**Affiliations:** ^1^Research Centre for Environment and Human Health, School of Continuing Education, Hong Kong Baptist University, Kowloon, Hong Kong SAR, China; ^2^College of International Education, Hong Kong Baptist University, Kowloon, Hong Kong SAR, China; ^3^Multidisciplinary Research Centre, School of Continuing Education, Hong Kong Baptist University, Kowloon, Hong Kong SAR, China; ^4^Institute of Bioresources and Agriculture, Hong Kong Baptist University, Kowloon, Hong Kong SAR, China; ^5^Division of Nursing Education, School of Continuing Education, Hong Kong Baptist University, Kowloon, Hong Kong SAR, China; ^6^Department of Biology, Hong Kong Baptist University, Kowloon, Hong Kong SAR, China

**Keywords:** COVID-19 pandemic, virtual nature contact, mental health, nature connectedness, restorativeness

## Abstract

The COVID-19 pandemic and its associated uncertainties and restrictions have adverse impacts on university students' mental wellbeing. Evidence shows that virtual nature contact has mental health benefits. However, little is known about the potential beneficial health impacts of virtual nature contact during times of social distancing, when access to the natural environment is restricted. This pilot study aimed to examine the effectiveness of a 3-week virtual nature contact in improving nature connectedness and reducing psychophysiological stress. A sample of 56 university students in Hong Kong was randomly assigned to control and nature interventions using 2-D video played for 15 min three times a week for 3 weeks. Nature connectedness, perceived restorativeness and psycho-physiological wellbeing were measured. Our findings show significant changes in psychological stress levels after nature interventions compared with the baseline, including increased happiness and stronger emotions of comfort and relaxation. When compared with the control group, the results show the nature intervention group has significantly higher levels of nature connectedness, happiness, and positive affect, but no significant effects on other psychological and physiological variables (e.g., cardiovascular responses). Our preliminary findings highlight the potential use of virtual nature contacts in bolstering university students' wellbeing at times of pandemic or when in-person visit to the natural environment is not feasible.

## 1. Introduction

The COVID-19 pandemic poses an unprecedented threat to global health. People experience distress from contracting this highly contagious virus, and pandemic health-control measures and the economic impact of the pandemic have an adverse impact on mental health (1). In the first year of the pandemic, the Global Burden of Disease 2020 study reported a 27.6% increase in the prevalence of major depressive disorder and a 25.6% increase in the prevalence of anxiety (2). As revealed in a meta-analysis, there is increasing concern about the general population's mental health during the COVID-19 pandemic. That analysis showed that the prevalence of stress was approximately one-third (29.6%) in a sample of 9,047 people from five studies, the prevalence of anxiety was 31.9% in a sample of 63,439 people from 17 studies, and the prevalence of depression was 33.7% in a sample of 44,531 people from 14 studies (3). In Hong Kong, young adults were particularly susceptible to stress, with approximately half (46%) of the population aged 18–24 reported feeling stressed, compared with less than one third (31%) of the general population (4). Another study's findings revealed that age as a risk factor for stress was further magnified during the COVID-19 pandemic, as young people across 26 countries and areas experienced higher levels of stress than older people (5). According to a territory-wide epidemiological study on youth mental health in Hong Kong (6), among 594 participants aged 15–24, 12% reported moderate to severe levels of stress. Overall, 22.8 and 22.5% of that study's participants reported moderate to severe levels of depression or anxiety symptoms, respectively. Fu et al. (7) reported that 41.1% of 89,588 college students showed anxiety symptoms. The COVID-19 pandemic and its associated uncertainties and restrictions on daily activities are damaging the mental health and wellbeing of adolescents. Another study shows that social distancing and social isolation reduced university students' perceived peer support, increased their perceived loneliness, and decreased their sense of hope, thus increasing their depressive symptoms (8). Werner et al. (9) reported that school lockdowns and closures increased loneliness and social stress and negatively impacted students' mental wellbeing. These findings have revealed an urgent need to address the overwhelmingly common stress problem in adolescents. There is a pressing need to develop effective stress interventions that are accessible to adolescents during the pandemic.

Stemming from Wilson's (10) biophilia hypothesis predicting the innate desire to connect with nature, a growing body of empirical research has cultivated staunch support for nature's benefits for mental and physical wellbeing (11–16). Li et al. (17) explored the link between exposure to nature and adolescents' moods in a real-life setting by recording the participants' locations for four consecutive days and matching the resulting data to their mood states. They revealed that increased exposure to nature throughout the day was significantly associated with decreased depression, anger, fatigue and overall negative mood. Another study suggested that the stress reduction property of nature works in two ways: it increases a person's distance from stressors and it restores a person's adaptive resources (18). In addition, attention restoration theory attempts to explain the mechanism of nature exposure in restoring human cognitive states (19, 20). One meta-analysis demonstrated that experiencing the components of nature requires little mental effort and hence can replenish a person's mental resources (21). A systematic review supported the benefits of interactions with nature in children and adolescents to their emotional wellbeing, stress, and overall mental health (22). Experimental evidence has also illustrated that nature exposure has promising results for improving mental wellbeing. Wilderness expeditions have been reported to be associated with improved self-esteem in adolescents (23, 24). Taking a nature walk significantly increases nature connectedness and positive affect compared to a control group in individuals with clinically diagnosed depression and anxiety (25). After a forest visit, the participants in another study reported lower levels of stress, fatigue, and irritation (26). Kotera et al. (27) compiled findings from six randomized controlled trials of forest-bathing and found the significant effects of forest-bathing on reducing depression and anger.

In the face of a threat, stress arousal contributes to activating a top-down mechanism from the brain to the body through the release of stress hormones, which elicit physiological responses, including cardiovascular functioning, to prepare the body to overcome threats (27). One study of laboratory stress tasks yielded results supporting the psycho-physiological nature of stress (28). Significant associations between perceived stress and increased heart rate, respiratory sinus arrhythmia, and cortisol levels were found in adolescents when performing a social stress task. Cardiovascular responses have also been well studied [e.g., Thayer et al. (29)]. It was reported that there is a positive association between stress and increased blood pressure that lasts even after the stressor disappears (30). Kim et al. (31) conducted a meta-analysis investigating how stress can be reflected in heart rate variability (HRV). The results from 37 studies consistently pointed to a significant change in HRV in response to stress, characterized by a decrease in the high frequency band and an increase in the low frequency band. With support from the literature, physiological changes, particularly cardiovascular responses, were deemed to be robust subjective measurements of stress. According to the stress recovery theory proposed by Ulrich and colleagues (32, 33), humans' physiology and psychology are evolutionarily adapted to the natural environment; thus, nature can relieve both physiological and psychological stress. Previous studies have demonstrated the benefits of spending time in nature for alleviating hypertension, reducing oxidative stress and stress hormones, and improving cardiac and pulmonary functions and emotional response (34). Furthermore, another study reported that a green schoolyard lowered students' physiological stress indicators, including blood pressure levels and HRV, and were associated with better mood and wellbeing (35). However, a systematic review of nature-based interventions on psycho-physiology stress recovery concluded that the evidence for physiological stress reduction with nature exposure is equivocal (36).

Recent research in nature-based intervention has turned the researchers' focus to the virtual nature experience, which is partly driven by the rapid urbanization of modern society, with an increasing number of people living in cities with limited nature nearby (37–39). According to the United Nations (UN), 55% of the world's population lives in urban areas, a percentage that is expected to increase to nearly 70% by 2050 (40). The increased accessibility of virtual natural environments *via* smartphones and virtual reality (VR) devices facilitates the virtual delivery of nature-based interventions (41, 42). Virtual nature provides nature exposure in an inexpensive, convenient, and less time-consuming manner than *in vivo* nature exposure (41, 43), and virtual nature can be experienced by individuals with mobility constraints (39). Browning et al. (42) reported that compared to a group of people who had an outdoor nature experience, a group that viewed short nature videos in VR experienced increased perceived restorativeness and positive mood. In addition, increasing research has explored the effectiveness of applying VR nature experiences in a wide range of healthcare applications, from pain management to depression (44–46). Valtchanov et al. (47) conducted a study with 22 participants and found a restorative effect of virtual forest immersion with VR akin to that of real-life nature exposure, resulting in increased positive affect and lower stress.

Unlike research into the benefits of virtual nature to ameliorate psychological stress, research into the benefits of virtual nature to ameliorate physiological stress has not yielded unambiguous results (48). The sound of nature can be crucial in recreating a dynamic natural setting for stress recovery (49, 50), as one group that experienced virtual nature with sound showed more parasympathetic activation than a group whose virtual nature experience did not include sound (51). Van den Berg et al. (52) illustrated that respiratory sinus arrhythmia, which is an indicator of parasympathetic activity, increased in a group of 46 students after they viewed photos of green spaces. However, a study with 30 participants failed to find a significant difference in systolic blood pressure and heart rate when comparing the effects of forest-environment and urban-environment VR experiences (48). Anderson et al. (53) concluded that there was no significant difference in objective physiological stress indicators, including electrodermal activity and HRV, after watching a 360° virtual nature video.

The prolonged COVID-19 pandemic and its associated prevention measures, such as citywide lockdowns, social-distancing rules, school closures, and working from home, have triggered mental and emotional stress and have limited people's ability to explore nature and experience green space *in vivo* (54). A virtual nature option would allow people who are unable to go outside to gain the benefits of nature exposure during the pandemic (55), and it might be an easily accessible alternative to *in vivo* nature exposure. However, there is a paucity of research examining the effects of virtual nature on the stress and health of adolescents during the COVID-19 pandemic (56). One preliminary study explored the plausibility of employing virtual nature as a stress-reduction intervention among COVID-19 frontline healthcare workers and found that the intervention had a significant stress-reducing effect (57).

To address the unmet need for an accessible and convenient stress-relief intervention amidst the COVID-19 pandemic, we aimed to conduct an exploratory study to examine the effect of virtual nature-based interventions on university students' psycho-physiological stress. Because our goal was to test an intervention that is easy to employ during the pandemic when social distancing measures were in place, we did not use specialized equipment or devices, but instead relied on a computer screen that played a 15-min 2-D video as an accessible method to obtain nature exposure at home. With most of the previous research adopting single interventions (42, 45–47, 55), we aimed to explore the effects of repeated nature exposure as a stress-reduction intervention package compared with control groups, with three types of virtual nature experience interventions conducted three times per week for three consecutive weeks. To explore the potential cumulative effects, follow-up assessments were conducted 2 weeks after the intervention period. When evaluating the effect of the virtual nature-based intervention, both physiological stress (particularly cardiovascular responses) and psychological stress were evaluated.

We hypothesized as follows: (a) Participants in the experimental group (virtual nature experience) will show lower levels of psychological and physiological stress responses after 3 weeks of interventions and during follow-up assessments compared with the baseline; and (b) Participants in the experimental group will show lower levels of psychological and physiological stress responses than the control group after 3 weeks of interventions and during follow-up assessments.

## 2. Materials and methods

### 2.1. Study design

Our study design involved a virtual experience-based pretest-posttest intervention with a control group. The independent variable was the virtual nature experience, whilst the dependent variables were the psychological and physiological health of university students. As part of a larger study investigating the influence of virtual nature exposure, this study aimed to examine the effects of a 3-week period of virtual nature experience on the psycho-physiological health.

### 2.2. Study population and sampling

Full-time students who were aged 18 years old or above and had the ability to comprehend our English-language questionnaire were recruited from a medium-sized university in Hong Kong. The exclusion criteria included self-reported mental illness, self-reported symptoms of disease, taking medicine, consuming caffeinated drinks and food, and consuming alcohol during the weeks of the study. Fifty-six participants were recruited, three of whom dropped out after registration due to other commitments, leaving an eligible sample of 53. All 53 participants completed the psychological measurements, and 28 participants agreed to the collection of data on the physiological stress variables. Because travel to the on-campus laboratory was required amidst the constraints of the fourth wave of COVID-19 in Hong Kong, we acquired a smaller sample of physiological stress variables than we would have acquired otherwise. The retention rate of the participants by the last week of the intervention was 84.5%. The study protocol was reviewed and approved by the Hong Kong Baptist University's Research Ethics Committee (REC/19-20/0306). All of the participants provided their prior informed consent. Convenience sampling was adopted through print advertisements and social media, including the school email network, Facebook, and Instagram, from December 2020 to November 2021. The participants were instructed to register and complete a demographic survey online. The eligible students who confirmed that they could attend all nine virtual nature experience sessions were then informed of arrangement of the interventions. To encourage retention, an incentive of HK$100 was offered to each participant who completed the study. The study was conducted between January and November 2021, during the fourth wave of COVID-19 pandemic, during which time Hong Kong had 12,436 confirmed cases (58), and 213 deaths as of 30 November 2021 (59). The Hong Kong government had adopted a dynamic zero-COVID policy, implementing strict anti-epidemic measures such as closing bars, schools, gym, cinemas, theme parks and sports venues; imposing a curfew for dine-in services; and adopting stringent travel restrictions.

### 2.3. Interventions and data collection procedure

**Figure 1:**
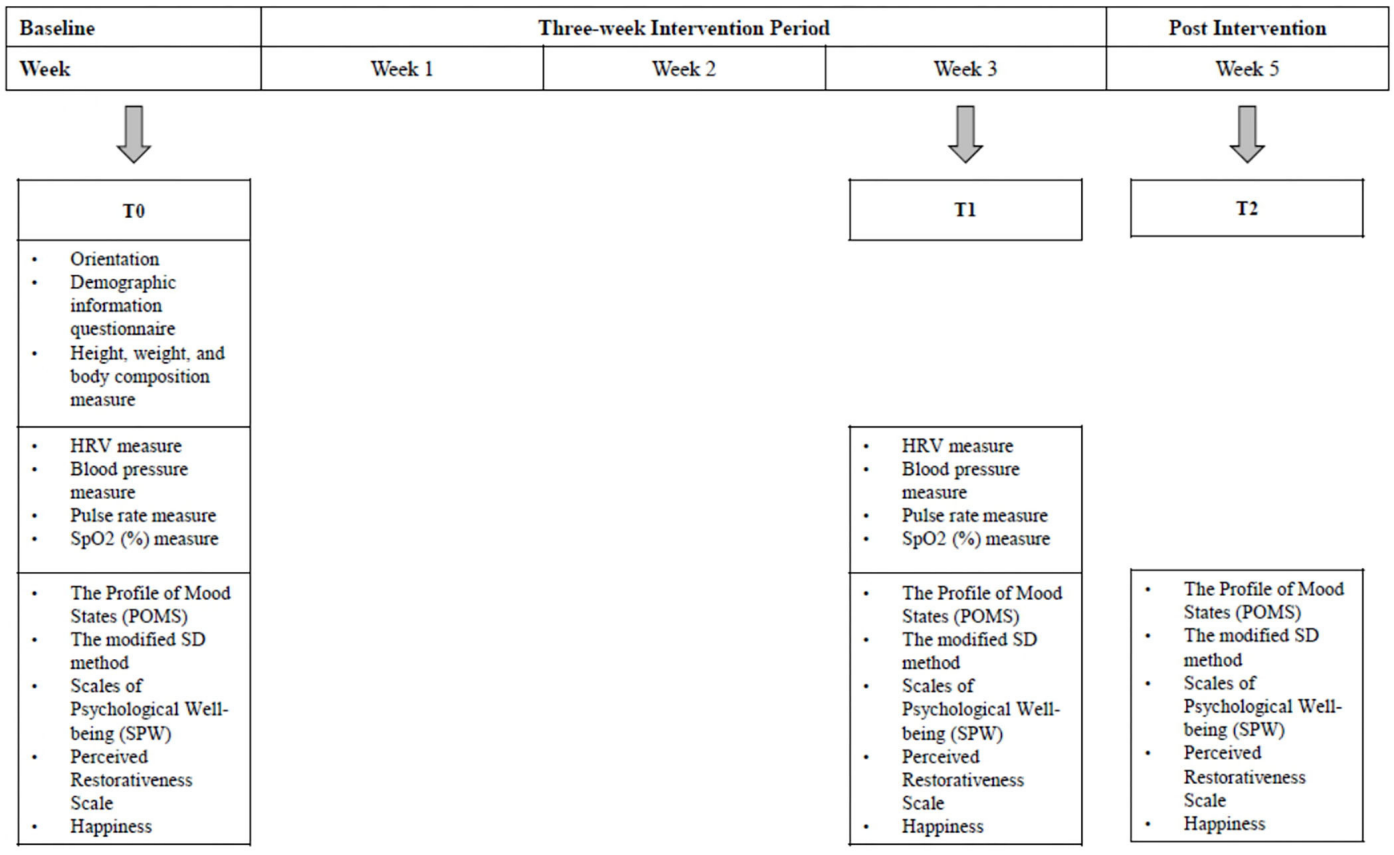
Measurements at different time points.

The participants were randomly assigned to different groups: the experimental groups, which included the urban nature (*n* = 11), marine nature (*n* = 9), and forest nature (*n* = 11) groups; and the control groups, including a shopping mall (*n* = 9) and a city (*n* = 13). A 15-min video was the medium for delivering the virtual experience of the two groups. The videos were collected from various public sources using YouTube (www.youtube.com/). To ensure that the videos could be used without copyright limitations, we selected those labeled for non-commercial reuse only. Each exposure condition included sounds but lacked a narrative about the conditions depicted. Figure 1 shows the procedure of the interventions and the measurement undertaken at different time points. To assess the cumulative effects, the interventions were administered three times per week for 3 weeks, giving nine interventions in total. Each intervention lasting for 15 min was adopted which was about the lower end of the intervention duration (20 min) recommended by Coventry et al. (60). One-way blinding was employed to minimize bias; thus, the participants were not informed of which group they would be assigned to before the study. During the first intervention session, the participants watched the clips corresponding to the randomized groups. Before watching the videos, the participants were instructed to take 3 min of active rest to minimize the impact of commuting or other disturbances. After the active rest, an HRV monitor, oximeter, and blood pressure monitor were placed onto the participants, and remained in place throughout the intervention. Next, the videos corresponding to the participants' assigned groups were played. Afterwards, post-test measurements were taken. Before first intervention (T0), after the nine interventions (T1), 2 weeks after the nine interventions (T2), the participants were asked to complete a questionnaire measuring their psychological responses. Noting the potential sleeper effect [i.e., a delayed effect after discontinuing the intervention (61)], T2 was measured with an aim to investigate the retention effects of the intervention. The physiological measurements were as follows: shopping mall: *n* = 5; city: *n* = 5; urban nature *n* = 5; marine nature *n* = 6; forest nature *n* = 7.

### 2.4. Measurement and instruments

A survey was used to collect data on the participants' demographic factors, including age and gender. The participants' psychological and physiological responses to the interventions were measured. A pilot study of eight participants was conducted on the logistics of the interventions, the physiological measurement, and the readability and clarity of the questionnaire to improve the administration and implementation of the main study.

#### 2.4.1. Psychological instruments

A self-reported questionnaire in English, which was approximately 15 min long, was administered to measure the participants' nature connectedness, stress, mood and psychological wellbeing.

**Degree of Nature Connectedness**. The degree of nature connectedness was measured by asking the respondents to indicate their degree of nature connected between themselves and nature using a score from zero to 100. The lowest self-reported degree of connectedness was represented by a score of zero, whereas a score of 100 indicated the highest degree of connectedness.

**Profile of Mood State (POMS)-Short Form**. Mood change was measured by the short form of POMS (62). The 37-item POMS was validated with a good internal consistency (Cronbach's α = 0.76–0.95) (63); it presents respondents with various adjectives regarding mood states and asks them to rate how well the adjectives describe their mood on a five-point Likert scale (0 = not at all, 4 = extremely well). On this scale, psychological distress is categorized into six dimensions—tension, depression, anger, vigor, fatigue, and confusion—creating six subscales. The depression subscale is measured using eight items in this 37-item short form, with items including unhappy and blue. The vigor and confusion subscales are measured using six and five items, respectively, including items such as lively and active for vigor and confused and forgetful for confusion. Whilst the tension subscale consists of six items, including tense and on-edge, the anger subscale contains seven items, for example, angry and peeved. Finally, the fatigue subscale consists of five items, including worn-out and weary. The values of all of the items are added into a total mood score, with a lower score indicating a more stable mood profile. The Cronbach's α of the present study were 0.94, 0.96, and 0.97 at baseline, week 3 and week 5, respectively.

**The Modified Semantic Differential (SD) Method**. The modified SD method (64) was used to evaluate participants' subjective emotions. The respondents were asked to evaluate their degree of emotion on a spectrum with semantically bipolar adjectives, for example, “comfortable–uncomfortable.” Three sets of opposing words were included, “comfortable–uncomfortable,” “natural–artificial,” and “relaxing–awakening.” Items are rated on a 13-point scale ranging from −6 (*very artificial*) to +6 (*very natural*), with a higher score indicating more positive emotional conditions.

**Scale of Psychological wellbeing (SPW)**. The SPW is a validated instrument derived from Ryff's model of psychological wellbeing (65) which contains six core dimensions, including autonomy, environmental mastery, personal growth, positive relations with others, purpose in life, and self-acceptance. The SPW has a good internal consistency (Cronbach's α = 0.86–0.93), test–retest reliability (Cronbach's α = 0.81–0.85), and good validity (65). The scale includes 18 items, with each item evaluated using a 7-point Likert scale (1 = *strongly disagree* to 7 = *strongly agree*). Each subscale consists of three items. Questions 1, 2, and 5 measure self-acceptance, whilst questions 3, 7, and 10 measure purpose in life. Items measured on the Personal Growth subscales are found in questions 11, 12, and 14, whilst questions 6, 13, and 16 measure items on the Positive Relation with Others subscales. Questions 15, 17, and 18 belong to the Autonomy subscale, and questions 4, 8, and 9 belong to the Environmental Mastery subscale. Questions 1, 2, 3, 8, 9, 11, 12, 13, 17, and 18 require reverse scoring, as the statements are oppositely worded to the direction of the subscale. The recording formula is the number of scale points plus one, minus the answer of the respondent. For example, if the participant answers 3 *(agree a little*) to Question 1, the recording formula is 7 + 1 – 3 = 5. The subscale scores are calculated by summing the response values of the items corresponding to the subscales. A higher score indicates better psychological wellbeing. The Cronbach's α of the present study were 0.81, 0.85, and 0.85 at baseline, week 3 and week 5, respectively.

**Perceived Restorativeness Scale (PRS)**. The PRS, developed by Hartig et al. (66), is a 26-item self-report validated scale for assessing the restorative quality of a particular environment. The scale was later shortened by Pasini et al. (67) to 11 items that capture factors such as Fascination, Being Away, Coherence, and Scope. Participants are asked to rate how well the statements describe their experience, on a 11-point scale, ranging from 0 (not at all) to 10 (completely/very much). Each factor was measured by three items, other than the Scope subscale, which was measured by two items only. An example of a Fascination subscale item is “In places like this it is hard to be bored.” Items on the Being away subscale include “Places like that are a refuge from nuisances”, “In places like this it is easy to see how things are organized” and “That place is large enough to allow exploration in many directions” are the example items for the Coherence and Scope subscales, respectively. A higher mean score indicates a higher level of perceived restorativeness. The scale is invariant across nationality and gender, suggesting good validity (67). The reliability of the short version of the PRS was previously reported as Cronbach's α = 0.79 (68). The Cronbach's α of the present study were 0.92, 0.94, and 0.91 at baseline, week 3 and week 5, respectively.

**Happiness**. To evaluate the participants' happiness, we used a single item scale (69) that has been shown to be a valid and reliable tool. The participants were asked to indicate how happy they felt at the moment on an 11-point scale ranging from 0 (not happy) to 10 (very happy).

#### 2.4.2. Physiological measurements

The average pulse rate, systolic blood pressure, and diastolic blood pressure were measured using a wireless portable blood pressure monitor (OMRON Smart Elite+). The average peripheral oxygen saturation (SpO2) was measured with an oximeter (O2Ring, US). In addition to the physiological indicators at a single time point, we collected data on HRV, which is a measure of the cardiac control exerted by the central autonomic nervous system (ANS) and a biomarker for stress for its association with emotional regulation ability (29). Various studies have demonstrated that HRV is significantly increased in response to stress in healthy participants (31). The converging evidence provides a strong basis for using HRV as an indicator of psychological stress. Therefore, we measured the participants' HRV using a wireless wearable HRV monitor (Polar H10 Heart Rate Sensor). HRV was represented by RMSSD, which is the square root of the mean of the sum of the squares of difference between adjacent to normal and normal intervals (31). The automatically generated Baevsky's stress index from the HRV monitor device was also used. This index is a geometric measurement of HRV, in which higher values indicate reduced variability and higher sympathetic cardiac activation, and hence higher cardiovascular system stress (70).

### 2.5. Statistical analysis

Our statistical analyses were performed using SPSS Statistical Package version 27 (SPSS Inc., Chicago, IL, USA). Descriptive statistics of the participants' demographics were calculated. The data were checked for normality by evaluating skewness, kurtosis, histogram, and normal plot, all of which confirmed a normal distribution. Three missing values were found to be missing at random with no outliers. The three experimental groups, namely, the urban nature group, marine nature group and forest nature group, were combined into the virtual nature group, whilst the two control groups, namely, the shopping mall group and city group, were combined into the urban group. Baseline data were compared between groups to detect the baseline difference. Paired sample *t*-tests were performed to compare the changes in psycho-physiological stress responses after 3 weeks of virtual nature experiences to the baseline in the nature group. In addition, to remove the placebo effect, stress responses after 3 weeks of virtual nature experiences were compared with stress responses in the control groups using an independent sample *t*-test. The assumptions of both tests were checked prior to the analyses. *P*-values of < 0.05 were used as the threshold to reject the null hypothesis.

## 3. Result

Among the total sample of 53 participants (73.6% female), the mean age was 20.3 years (SD = 1.2). The mean scores and standard deviations of the participants' psychological and physiological stress are reported in Tables 1, 2, respectively. None of the psycho-physiological stress variables showed significant differences between the virtual nature group and the urban group at the baseline, as revealed by the independent *t*-test, *p* > 0.05.

**Table 1:** Mean scores and standard deviations of the psychological stress of the virtual nature group and urban group at baseline (T0), week 3 (T1), and week 5 (T2).

	**Virtual nature group**			**Urban group**		

	**T0**	**T1**	**T2**	**T0**	**T1**	**T2**
**Psychological stress variables**						
Nature connectedness	67.1 (18.6)	74.9 (14.9)	74.2 (16.2)	56.6 (19.9)	47.6 (22.4)	51.8 (23.8)
Total mood disturbance (POMS)	40.3 (23.3)	32.9 (24.8)	31.6 (24.3)	31.1 (19.5)	35.9 (21.5)	36.2 (25.4)
Perceived restorativeness (PRS)	19.8 (4.9)	21.3 (4.3)	21.1 (3.8)	19.9 (4.5)	18.3 (5.9)	17.8 (5.2)
Happiness	6.7 (2.1)	7.7 (1.9)	7.9 (1.5)	7.6 (1.60)	7.3 (2.0)	7.4 (1.8)
Psychological well-being (SPW)	84.0 (13.1)	84.1 (15.4)	88.9 (16.5)	84.9 (11.5)	84.7 (10.8)	83.6 (11.7)
Degree of emotion: comfortable to uncomfortable	8.2 (2.5)	9.2 (2.4)	8.9 (2.5)	8.4 (2.7)	7.6 (2.7)	8.1 (2.3)
Degree of emotion: relaxed to aroused	8.3 (1.8)	9.4 (2.3)	8.47 (2.55)	7.8 (2.7)	7.8 (2.4)	8.0 (1.8)
Degree of emotion: natural to artificial	7.7 (2.4)	9.6 (1.9)	8.9 (1.8)	6.9 (3.0)	6.0 (2.5)	7.1 (2.5)

**Table 2:** Mean scores and standard deviations of the physiological stress variables in the virtual nature group and urban group (*N* = 28).

	**Virtual nature group**		**Urban group**	

	**T0**	**T1**	**T0**	**T1**
Average SpO_2_ (%)	97.3 (0.6)	97.2 (0.65)	97.3 (0.7)	97.4 (0.8)
Average pulse rate	75.3 (11.1)	78.9 (11.4)	78.1 (12.8)	81.2 (13.9)
RMSSD	43.1 (22.2)	65.1 (87.4)	41.6 (23.1)	43.5 (47.3)
Stress index	10.3 (4.8)	8.9 (4.8)	11.0 (5.8)	11.2 (4.7)
Systolic blood pressure	−1.0 (10.7)	2.2 (10.7)	−1.4 (14.7)	4.6 (10.5)
Diastolic blood pressure	−2.4 (6.8)	−3.7 (8.6)	−1.8 (10.7)	0.6 (11.0)

### 3.1. Effects of the interventions on the participants' psychological and physiological stress over time

It was hypothesized that participants in the virtual nature-based interventions would show improved psychological and physiological stress compared to the baseline. A paired *t*-test was conducted to compare the effects of the 3 weeks of virtual nature interventions on the participants' psycho-physiological responses with the baseline. The assumptions of the paired *t*-test were not violated. Table 3 summarizes the results of the paired and independent *t*-tests.

**Table 3:** Between-subject and within-subjects comparison of the psycho-physiological stress variables at T0, T1, and T2.

	**Independent** ***T*****-tests**			**Paired** ***T*****-tests**	

	**T0**	**T1**	**T2**	**T0–T1**	**T0–T2**
**Psychological stress variables (*****n*** = **53)**					
Nature connectedness	*t*_(51)_ = −1.95	*t*_(45)_ = −5.01 ^***^	*t*_(33)_ = −3.31^**^	*t*_(25)_ = −2.29^*^	*t*_(18)_ = −1.17
Total mood disturbance (POMS)	*t*_(51)_ = −1.53	*t*_(45)_ = 0.43	*t*_(33)_ = 0.55	*t*_(25)_ = 1.62	*t*_(18)_ = 2.19^*^
Perceived restorativeness (PRS)	*t*_(51)_ = 0.08	*t*_(45)_ = −1.97	*t*_(33)_ = −2.20 ^*^	*t*_(25)_ = −1.46	*t*_(18)_ = −1.86
Happiness	*t*_(51)_ = 1.68	*t*_(45)_ = −0.66	*t*_(33)_ = −0.81	*t*_(25)_ = −2.34^*^	*t*_(18)_ = −3.31^**^
Psychological wellbeing (SPW)	*t*_(51)_ = 0.25	*t*_(45)_ = 0.15	*t*_(33)_ = −1.07	*t*_(25)_ = −0.19	*t*_(18)_ = −1.48
Degree of emotion: comfortable to uncomfortable	*t*_(51)_ = 0.24	*t*_(45)_ = −2.17^*^	*t*_(33)_ = −1.02	*t*_(25)_ = −2.11	*t*_(18)_ = −0.96
Degree of emotion: relaxed to aroused	*t*_(51)_ = −0.89	*t*_(45)_ = −2.33^*^	*t*_(33)_ = −0.63	*t*_(25)_ = −2.31^*^	*t*_(18)_ = −0.16
Degree of emotion: natural to artificial	*t*_(51)_ = −1.07	*t*_(45)_ = −5.73 ^***^	*t*_(33)_ = −2.45 ^*^	*t*_(25)_ = −3.07^**^	*t*_(18)_ = −1.33
**Physiological stress variables (*****n*** = **26)**					
Average SpO_2_ (%)	*t*_(26)_ = 0.09	*t*_(26)_ = 0.63		*t*_(17)_ = 0.27	
Average pulse rate	*t*_(26)_ = 0.60	*t*_(26)_ = 0.48		*t*_(17)_ = −0.85	
RMSSD	*t*_(26)_ = −0.17	*t*_(26)_ = −0.72		*t*_(17)_ = −1.05	
Stress index	*t*_(26)_ = 0.33	*t*_(26)_ = 1.20		*t*_(17)_ = 0.93	
Systolic blood pressure (before–after)	*t*_(26)_ = −0.08	*t*_(26)_ = 0.57		*t*_(17)_ = 0.74	
Diastolic blood pressure (before–after)	*t*_(26)_ = 0.20	*t*_(26)_ = 1.15		*t*_(17)_ = −0.48	

T0: Baseline, T1: Week 3, T2: Week 5.

* p < 0.05,

** p < 0.01,

*** p < 0.001.

**Figure 2:**
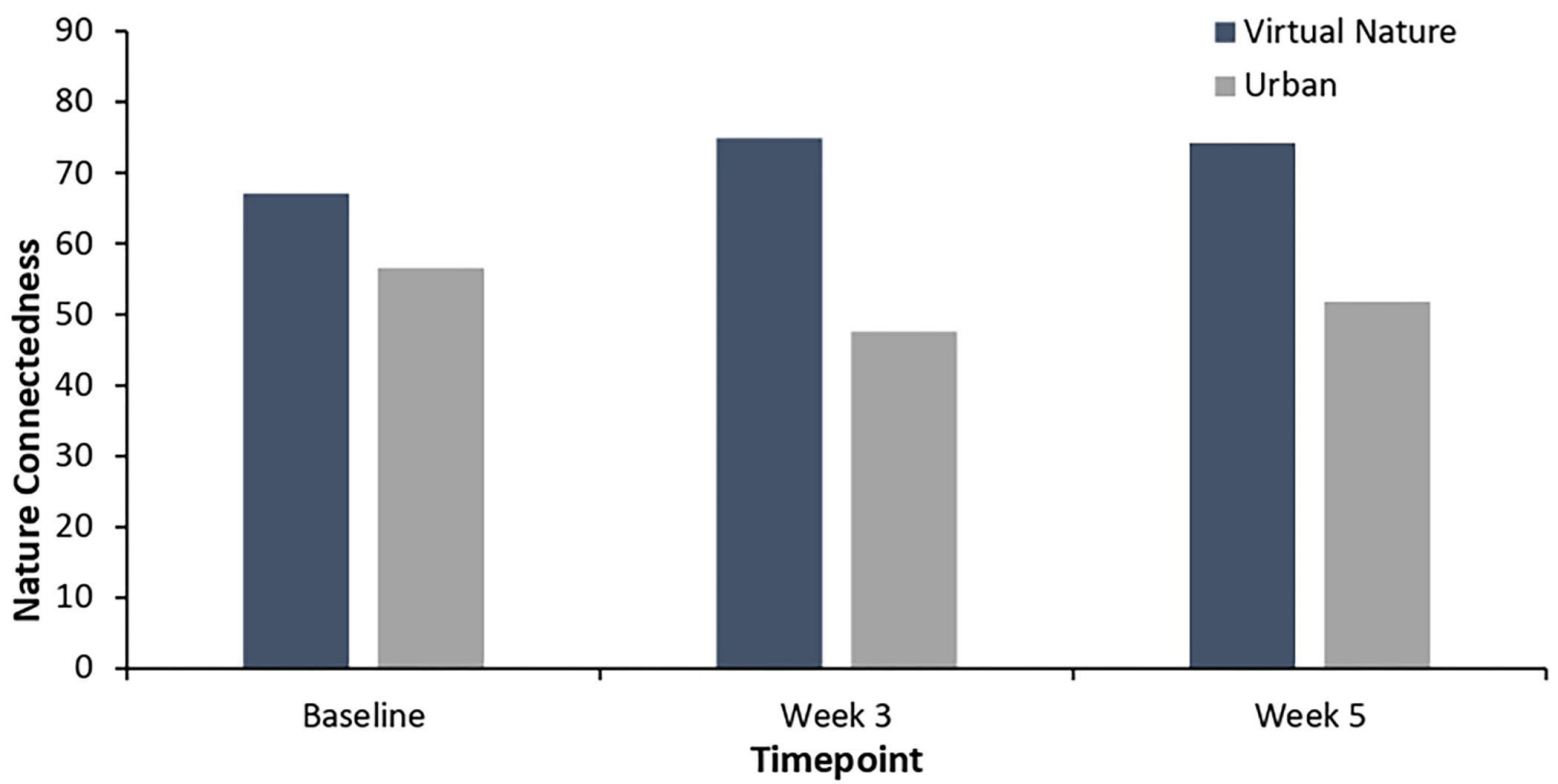
Nature connectedness in the virtual nature group and urban group from baseline to week 5. Levels of nature connectedness in the virtual nature group were higher than in the urban group in weeks 3 and 5. There was an increase in nature connectedness in weeks 3 and 5 over the baseline.

**Figure 3:**
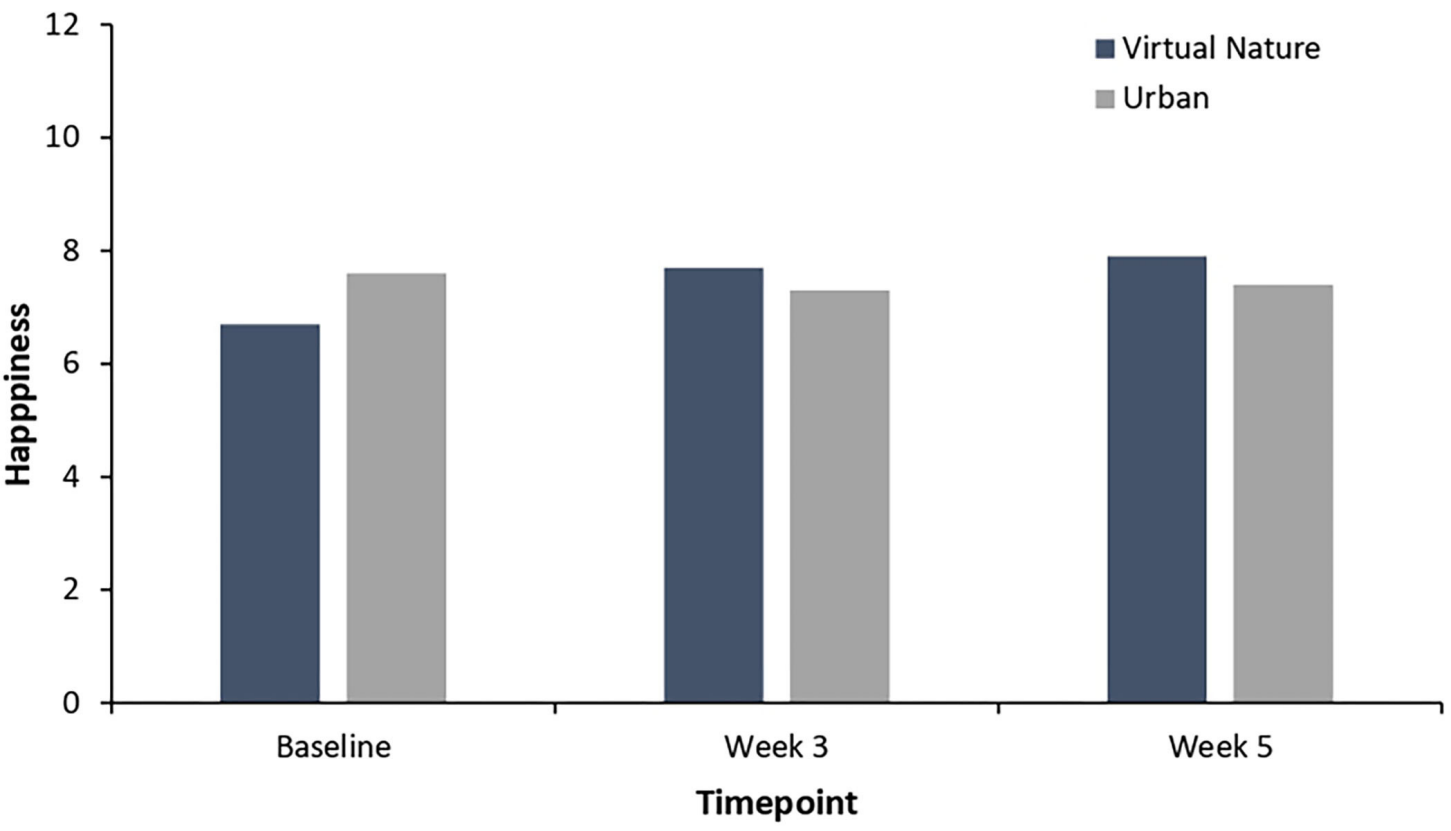
Happiness in the virtual nature group and urban group from baseline, week 3 and week 5. Levels of happiness in the virtual nature group were higher than in the urban group in week 3 and week 5. There was an increase in happiness in week 3 and week 5 over baseline.

**Figure 4:**
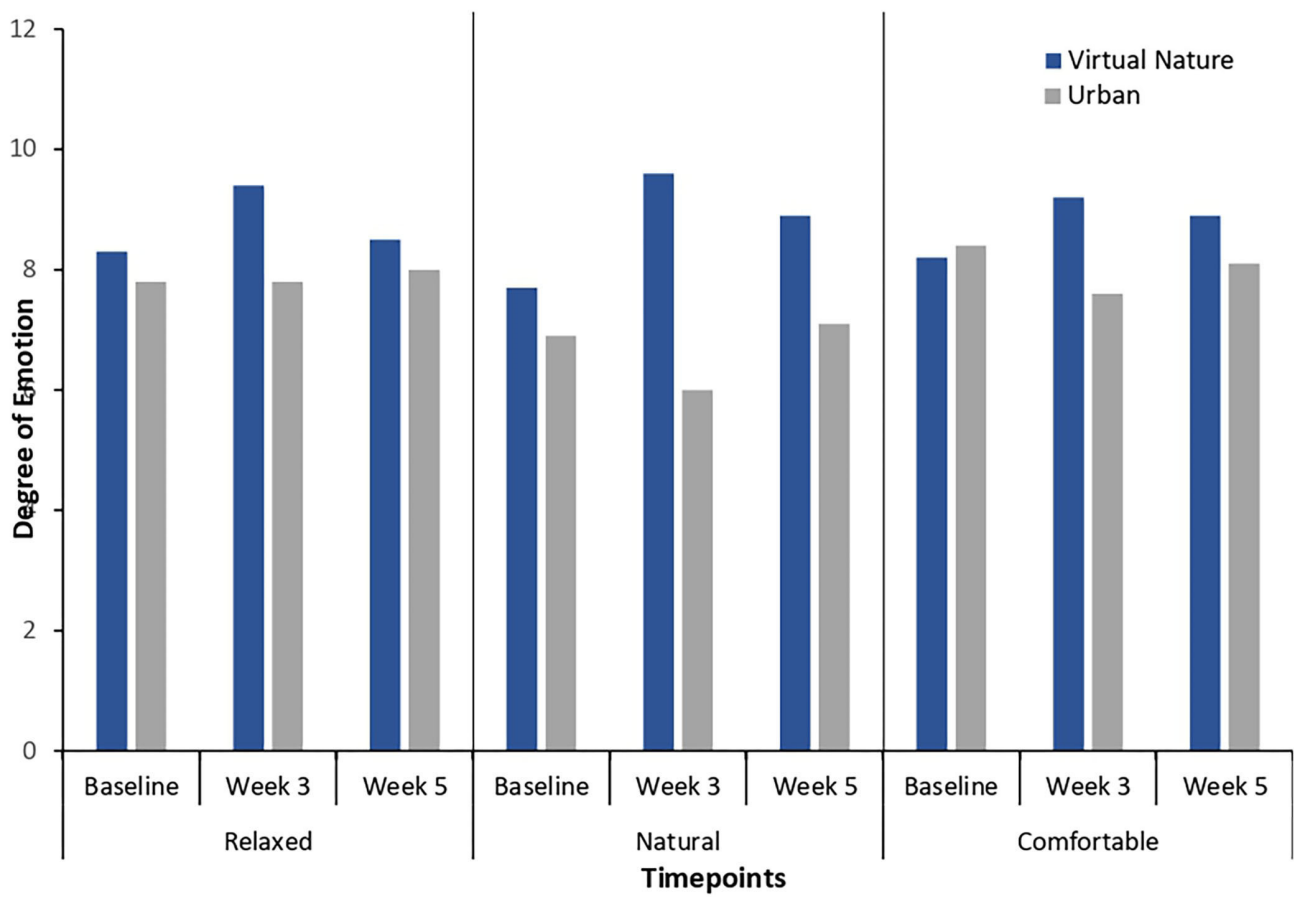
Degree of relaxed, natural, and comfortable mood at baseline (T0), week 3 (T1), and week 5 (T2) in the virtual nature group and urban group. The increase in relaxed, natural mood in week 3 from the baseline in the virtual nature group reached statistical significance. Although there was a trend of increase in comfortable mood in week 3 from the baseline, that change was not statistically significant. Moreover, the levels of relaxed, natural, and comfortable mood in the virtual nature group were higher than those in the urban group in week 3. Although the differences did not reach statistical significance, there was a greater trend of a more relaxed and comfortable mood in the virtual nature group than in the urban group at the week 5.

As shown in Figure 2, the participants in the virtual nature group showed significantly higher levels of nature connectedness after 3 weeks of intervention than the baseline, *t*_(25)_ = −2.29, *p* = 0.03. In addition, the participants in the virtual nature group reported significantly higher levels of happiness at week 3 than the baseline, *t*_(25)_ = −2.34, *p* = 0.03, as depicted in Figure 3. Significant increases over the baseline in terms of the degree of relaxed and natural emotions at week 3 were also found as follows: relaxed: *t*_(25)_ = −2.31, *p* = 0.03; natural: *t*_(25)_ = −3.07, *p* = 0.005. These results are further visualized in Figure 4. However, the degree of comfortable emotions, total mood disturbance, perceived restorativeness, and psychological wellbeing in the virtual nature group did not show significant differences at Week 3 compared to the baseline, *p* > 0.05. Our analyses also revealed no significant difference in any of the physiological stress variables at week 3 compared to the baseline, *p* > 0.05.

### 3.2. Effects of the interventions on the participants' psychological and physiological stress vs. the urban group

To test the second hypothesis that the participants who received virtual nature experiences would show lower levels of psychological and physiological stress responses than the participants in the urban group, independent *t*-tests were conducted to compare the stress variables at Week 3 in the virtual nature group vs. the urban group. Levene's test of equality of variance suggested that the assumption of homogeneity of variance had not been violated, with *p* > 0.05 in all variables at Week 3. The assumptions for the independent *t*-test were met, and hence the results can be interpreted accurately.

**Figure 5:**
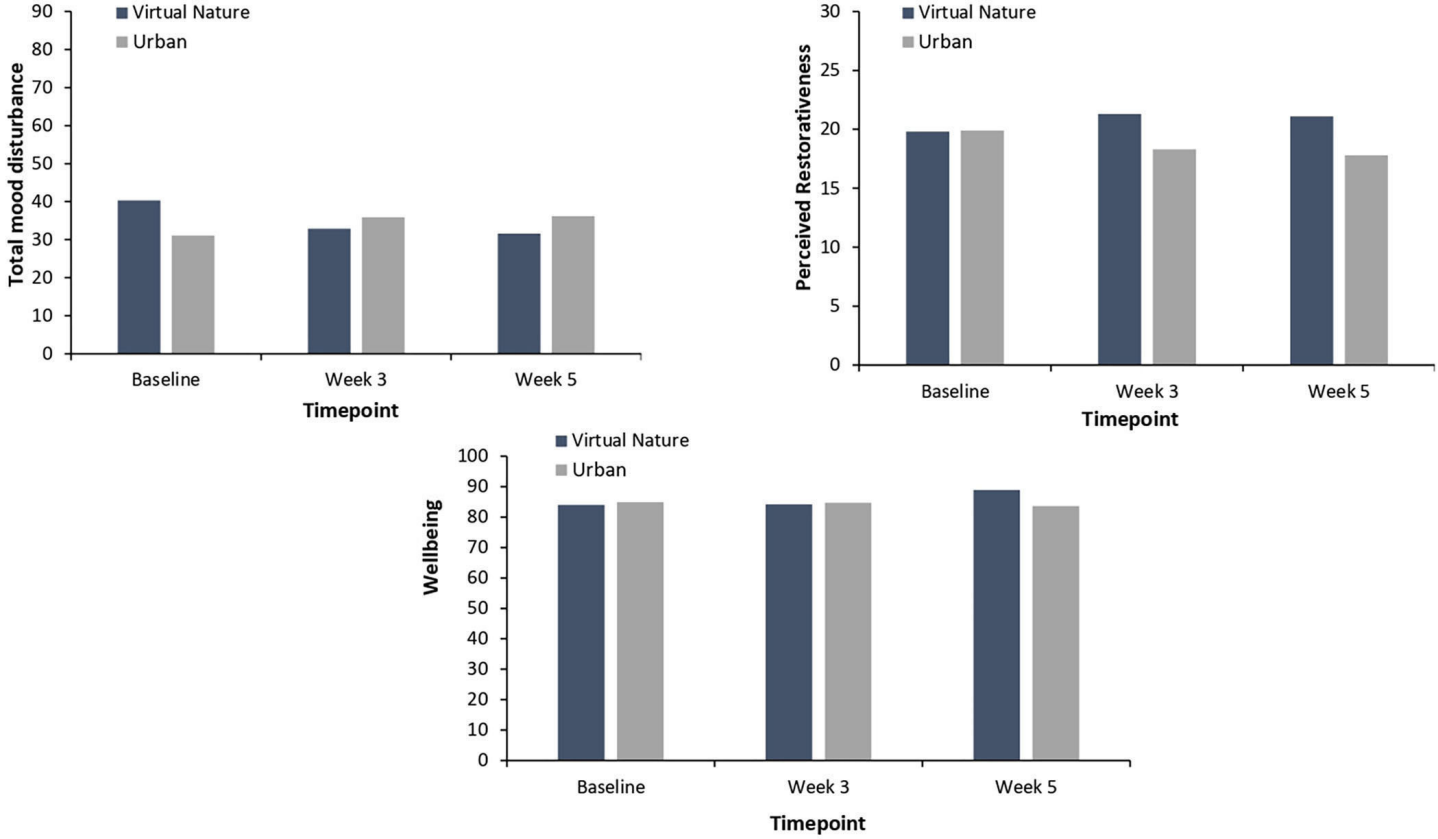
Levels of perceived restorativeness, total mood disturbance, and psychological wellbeing of the virtual nature group and urban group at baseline, week 3 and week 5. No significant difference was found between the groups in week 3 and the week 5. Moreover, no significant changes were found in either group between baseline and week 5.

Table 3 illustrates the results from the independent *t*-test comparing psycho-physiological stress in the virtual nature group with that in the urban group at Week 3 and at the follow-up. As seen in Figure 2, the participants reported significantly higher levels of nature connectedness after 3 weeks of virtual nature experience than the participants in the urban group did at Week 3: *t*_(45)_ = −5.01, *p* < 0.001. Additionally, as shown in Figure 4, a significantly higher degree of natural emotion was found in the virtual nature group than in the urban group by the end of Week 3, *t*_(45)_ = −5.73, *p* < 0.001. The degree of comfortable and relaxed emotions was also significantly higher in the virtual nature group than in the urban group after 3 weeks as follows: comfortable, *t*_(45)_ = −2.17, *p* = 0.04; relaxed, *t*_(45)_ = −2.33, *p* = 0.03. Despite the significant within-subject change in happiness shown in Figure 3, there was no significant difference between the groups at Week 3 and follow-up. However, as shown in Figure 5, for total mood disturbance, perceived restorativeness, and psychological wellbeing, the difference between the virtual nature and urban groups at Week 3 did not reach statistical significance, *p* > 0.05. None of the physiological stress variables measured at Week 3 showed significant differences between groups, *p* > 0.05.

### 3.3. Retention effects of the interventions on participants' psychological stress vs. the urban group

A follow-up assessment of the participants' psychological variables was performed 2 weeks after the intervention period. Compared to the baseline using paired *t*-tests, significant differences were found in happiness, *t*_(18)_ = −3.31, *p* = 0.004, and total mood disturbance, *t*_(18)_ = 2.19, *p* = 0.042. The increased degree of positive emotions, including comfortable, relaxed, and natural feelings at the end of the intervention, were not sustained 2 weeks after the intervention, *p* > 0.05. The participants' nature connectedness, perceived restorativeness, and psychological wellbeing reported at the 2-week follow-up did not significantly differ from the baseline, *p* > 0.05.

A comparison of the groups at follow-up was made using independent *t*-tests. Whilst Levene's test for equality of variance confirmed that the variables' variance did not differ significantly between the groups at the follow-up, *p* > 0.05, our assumptions were validated. When compared to those in the urban group, our results demonstrated that the heightened nature connectedness of the virtual nature group over the urban group remained during the follow-up, *t*_(33)_ = −3.31, *p* = 0.002, along with the increased emotions of feeling natural, *t*_(33)_ = −2.45, *p* = 0.02. The boost in degree of comfortable and relaxed emotions seen at the end of the 3-week intervention, however, was not sustained after 2 weeks, *p* > 0.05. Despite the absence of significant differences in perceived restorativeness at Week 3, the participants in the virtual nature group reported significantly higher levels of perceived restorativeness at the follow-up than at the baseline, *t*_(33)_ = −2.20, *p* = 0.04. Total mood disturbance, happiness, and psychological wellbeing at the follow-up did not significantly differ from the baseline.

## 4. Discussion

In light of the increased attention being paid to the mental health of adolescents during the COVID-19 pandemic, we aimed to evaluate the effect of a simple virtual nature-based intervention, i.e., watching a 2-D video, on reducing psycho-physiological stress in university students. This study provides evidence to add to the existing literature on the effectiveness of brief virtual nature-based intervention on the wellbeing of the university students during the pandemic. Our findings show that watching 15-min videos of three nature-based scenes, including urban nature, marine nature and forest nature, for 3 weeks resulted in psychological benefits for the participants, namely, increased happiness, along with comfortable, relaxed, and natural emotions. However, no influence was found on physiological stress. Despite increasing research into the adoption of VR technology in nature-based interventions that emphasize a fuller immersion in a simulated natural environment, we demonstrate that a simple 2-D video can be an effective, low-cost, convenient alternative to *in vivo* nature experiences during the pandemic.

### 4.1. Virtual nature and psychological stress

Previous studies have demonstrated that virtual nature delivered by various methods increases positive affect and reduces stress (46). Our findings are somewhat consistent with studies that have previously reported the hedonic effects of virtual nature exposure (42, 47), although support for a reduction in negative affect is less clear. Consistent with the recent literature, enhancements in positive affect and happiness were found after 3 weeks of the virtual nature-based intervention compared to the urban group and the baseline. Similarly, Keltner et al. (71) researched the emotional changes in study participants in six countries after they viewed short clips of natural history television content, and reported significant increases in positive feelings, including awe, contentedness, and joy; this change was significantly different from that observed in the control group. Whilst the literature has primarily adopted single viewings of nature videos, this study may add to the current understanding of the psychological effects of repeated viewings.

Our results showed that the intervention had no effect on psychological wellbeing as measured by SPW. Because Ryff's SPW measure of psychological wellbeing focuses heavily on eudaimonia, which is characterized by self-acceptance and fulfilling one's life purpose (65), the lack of change in psychological wellbeing might be attributable to the relatively constant nature of eudaimonia. In comparison to research that has successfully improved eudaimonic wellbeing (65), a 3-week intervention protocol may be too brief to exert effects on the participants' eudaimonia. Meanwhile, the participants' total mood disturbance only showed significant changes from the baseline during the follow-up, but not at the end of Week 3. Thus, this finding should be treated with great caution. Notably, because of the exploratory nature of this study, it included a more comprehensive group of variables than previous studies, allowing the measurement of multiple facets of psychological wellness. Given the multitude of outcome variables, this study had a higher likelihood of obtaining mixed results. Frost et al. (72) systematically reviewed 21 studies of the psychological effects of virtual nature immersion. They reported that 33% of the studies provided evidence supporting a reduction in negative effects, whilst 38% of the studies either failed to observe changes or observed an increase in negative affect. The evidence base for the psychological impact of virtual nature experiences remains inconclusive and warrants further research.

Coinciding with the significant improvement in positive mood, the experimental videos successfully cultivated strong nature connectedness in the participants during all weeks and at the follow-up. A study on 863 participants in China investigated the links between nature exposure, nature connectedness, and mental wellbeing (73) and found that nature connectedness moderated the associations between nature exposure, as measured by visitation frequency, nearby greenspace and park quantity, and mental wellbeing. Similarly, another recent study revealed that nature connectedness and nature restorativeness mediated the relationship between nature exposure and quality of life in 924 Lithuanians (74). The increase in nature connectedness over the control group and baseline following a virtual nature experience is consistent with the well-established evidence of how nature exposure affects mental wellbeing by eliciting nature connectedness. The concurrent increase in nature connectedness and positive affect provides primary support for increasing positive affect with 2-D videos that induce nature connectedness.

Nature exposure research has established the close association of perceived restorativeness and nature connectedness (75). A mediation analysis carried out by McAllister et al. (76) supported the proposition that perceived restorativeness is a mediator of nature experience and affect. However, despite the significant between-group difference in nature connectedness during Week 3 and at the follow-up in this study, the participants' perceived restorativeness only showed a significant difference at the follow-up. The reason for this discrepancy is unclear, one plausible explanation may be related to the spatial characteristics of the nature intervention. Tabrizian et al. (77) demonstrated that the permeability and arrangement of vegetation moderated the restorativeness of a green space by affecting the environment's perceived safety. A more dense and enclosed green space is associated with lower perceptions of safety and restorativeness. Therefore, future research in virtual nature should consider considering spatial characteristics which may influence the perception of the environment.

### 4.2. Virtual nature and physiological stress

Our findings suggest that 3 weeks of virtual nature exposure has no significant effect on any of the physiological variables. According to a systematic review by Frost et al. (72), 10 virtual nature studies that measured physiological indicators of stress, ranging from salivary cortisol to heart rate variability, returned mixed results. In line with our results, the blood pressure and HRV of that study's participants' did not display significant changes after watching 360° or VR videos of nature (48, 53). Nukarinen et al. (78) concluded that unlike their real-life nature group, the participants in their virtual nature group did not show significant restoration in terms of HRV and pulse rate. In contrast, some researchers have found evidence supporting the proposition that virtual nature experiences can lower a person's heart rate (79, 80) and HRV (51). For instance, Alyan et al. (79) conducted a study with 20 participants in Finland who were asked to go on virtual forest walks. They found a significant decrease in heart rate and skin conductance level, along with lower levels of depression and confusion in POMS. The nonsignificant findings of this study might be because the nature exposure was not immersive enough to translate psychological changes into physiological changes. Another possible explanation for the lack of significant changes in the physiological variables in the current study could be that the outcome measurements emphasized the physiological indicators of stress reductions, mainly cardiovascular stress responses. It could be argued that the psychological changes observed in this trial were mainly hedonic in nature, including an increase in happiness and positive affect, like comfortable and relaxed feelings. Psychosomatic research suggests that happiness and joy can also increase blood pressure (81), which is inconsistent with reduced blood pressure during relaxation. Notwithstanding the well-established evidence of real-life nature exposure on physiological stress, the physiological influence of virtual nature remains inconclusive.

## 5. Study limitations and future research

Although the current exploratory research provided some preliminary findings regarding the benefits of virtual nature experience on nature connection and health, the study has several limitations. First, there may have been a selection bias in the sampling, as students who were either more stressed or more interested in nature experiences than others were more likely to participate in the study. However, an incentive of $100 HKD (i.e., $12.78 UDS) would be able to attract students to participate regardless of their stress levels or interest level in nature experience. Noting from a large population study of 4,960 adults in England by Martin et al. (82) with the findings showed that trait connectedness to nature moderated the relationships between nature contacts and psychological wellbeing, future studies could consider measuring participants' personal inclination to connect with nature, e.g., how often they visit nature. Second, there was an imbalance amongst the virtual nature group and the urban group in that the numbers of controlled participants did not match those in the intervention groups. An unequal sample size of the two groups might have a different probability of resulting in type one or two errors (83). Third, it should be acknowledged that the sample size is small for a conclusive interpretation of the interventions' effects. Future research is required to replicate the study with a larger sample size, and with equally sized control and experimental groups, to capture the effectiveness of the virtual nature experience with a higher degree of confidence. Fourth, the participants' preference for natural environments was not measured in the study. Other research showed a significant correlation between the participants' appreciation of various natural environments and the improvement in their mood after experiencing them in VR (84). Future trials could take this into consideration to enhance the experience. Fifth, in the current study design, the content of the videos in each group was the same throughout the 3-week intervention period. It is unclear whether the repetition might have caused the participants to become bored with or tired of the intervention. The evidence suggests that there is a positive association between the psycho-physiological benefits of nature exposure and species richness and habitat diversity (85). Further research is needed to explore whether videos of various natural environments, or videos of various scenes of the same natural environments, can be used to maximize the participants' perception of biodiversity. Sixth, this study focused on the results of pre-post-intervention comparison of a 3-week virtual nature experience only, but without investigating the continuous changes of the physiological factors (such as cardiovascular variables) which were rather fluctuated as expected. The fuller picture of the benefits of the virtual nature contact could be further explored in future studies when the continuous changes of these variables are investigated. Seventh, because the intervention was self-administered in the participants' homes due to the COVID-19 pandemic and university campus closures, the above-referenced fluctuations are likely to have been influenced by other confounding factors that are unaccounted for, warranting future research. Future studies could randomize the control group and experimental group with participants of balanced gender according to the levels of participants' personal inclination to connect with nature in order to reduce the potential bias.

## 6. Implications

The COVID-19 pandemic has brought a rapid increase in stress, and psychopathology such as anxiety and depression (2, 3). Smith et al. (86) compared the difference of nature relatedness between actual nature and virtual nature experiences and found no significant differences between them. As technology advances, researchers have adopted different methods to deliver virtual exposure to nature, including 2-D videos, 360° VR viewing, and interactive computer-generated VR. However, there has been only limited research into how the effectiveness of these stimulations vary. Yeo et al. (87) compared the effectiveness of these three virtual nature delivery modes for lowering negative affect and increasing positive affect and found that despite the fact that interactive computer-generated VR provided the highest level of nature connectedness and increase in positive affect, all of the conditions achieved a significant reduction in negative affect that did not differ between the simulation forms. As three-dimensional VR technology becomes prevalent, more research studies have employed VR than have employed 2-D videos as the medium to deliver nature exposure (42, 44, 88). Nonetheless, the potential benefits of a more accessible and generalisable form of virtual nature should not be undervalued when developing stress reduction interventions with clinical implications.

This study demonstrates that with the objective of exploring an accessible method to obtain regular nature exposure during the pandemic that requires no special equipment to self-administer, watching 2-D videos of nature could be an adequate form of delivery. Samus et al. (89) reported that in 261 participants, having a private garden with high biodiversity predicted strong nature connectedness, which in turn predicted more positive emotions during New Zealand's 2020 COVID lockdown. Hence, maintaining a good level of nature connectedness is crucial to maintaining wellbeing during stressful times or when mobility is limited. The implications of the restorative effects of virtual nature have been explored in patients with chronic pain, cancer, and dementia (44). Promising results were found in a pilot study of nature-based VR experiences on 24 healthcare professionals during COVID-19 in the United States (90), with a significant alleviation of emotional distress and boost in focus reported.

## 7. Conclusions

Our exploratory study provides preliminary findings in supporting the positive effect of a 3-week virtual nature experience on nature connectedness, happiness, and positive affect during the COVID-19 pandemic. The need to facilitate virtual nature contact opportunities for youth is underscored during times of crisis, when outdoor nature-based activities are limited. Virtual nature contact might be a simple self-help tool for stress relief that does not require a visit to an outdoor natural environment nor specialized equipment like VR. Future studies with larger samples that make comparisons with real-life nature exposure are warranted to validate the effectiveness and clinical significance of virtual nature experience interventions to target stress reduction.

## Data availability statement

The raw data supporting the conclusions of this article will be made available by the authors, without undue reservation.

## Ethics statement

The studies involving human participants were reviewed and approved by Hong Kong Baptist University's Research Ethics Committee. The patients/participants provided their written informed consent to participate in this study.

## Author contributions

Conceptualisation and study design, coordination of the study, and funding requisition: SLa. Development of the study methodology: SLa, SLe, JoW, TL, and SC. Data collection: SLa, JM, JaW, and EC. Data analysis: SLa, JM, EC, and RC. Writing up of the manuscript: SLa and RC. All authors have read and agreed to the published version of the manuscript and review of the manuscript.
