# Supplementary material for: Brief repeated virtual nature contact for three weeks boosts university students' nature connectedness and psychological and physiological health during the COVID-19 pandemic: A pilot study
Source: Front Public Health. 2023 Jan 12;10:1057020. doi: 10.3389/fpubh.2022.1057020 (PMC9878182; doi:10.3389/fpubh.2022.1057020)
Supplement: Supplementary file 1 [file Table_1.docx]

**Supplementary information**

Subscales of the Profile of mood states (POMS) and Perceived restorativeness scale (PRS) of the Virtual Nature Group and Urban Group at Baseline (T0), Week 3 (T1), and Week 5 (T2)

|  | Virtual Nature Group  (n = 31) | | | Urban Group  (n = 22) | | |
| --- | --- | --- | --- | --- | --- | --- |
|  | T0 | T1 | T2 | T0 | T1 | T2 |
| **POMS** (M(SD)) | 40.3  (23.3) | 32.9  (24.8) | 31.6  (24.3) | 31.1  (19.5) | 35.9  (21.5) | 36.2  (25.4) |
| - Depression (8 items) | 14.1  (6.6) | 11.8  (6.2) | 11.7  (6.2) | 12.6  (4.7) | 12.5  (5.9) | 12.5  (5.8) |
| - Vigor (6 items) | 15.4  (6.1) | 15.3  (5.8) | 16.1  (5.9) | 17.6  (5.4) | 16.4  (5.4) | 16.6  (4.8) |
| - Confusion (5 items) | 10.9  (4.5) | 8.7  (4.9) | 8.4  (4.4) | 9.4  (3.0) | 9.7  (4.0) | 9.1  (4.1) |
| - Tension (6 items) | 11.2  (5.4) | 9.5  (6.2) | 9.7  (5.3) | 9.7  (4.0) | 10.8  (5.0) | 10.4  (5.1) |
| - Anger (7 items) | 10.1  (4.5) | 10.3  (6.2) | 9.4  (4.5) | 9.0  (3.3) | 9.8  (4.2) | 11.3  (5.9) |
| - Fatigue (5 items) | 9.5  (4.2) | 7.8  (4.2) | 8.5  (4.9) | 8.2  (3.8) | 9.5  (4.6) | 9.5  (4.4) |
| **PRS** (M(SD)) | 19.8  (4.9) | 21.3  (4.3) | 21.1  (3.8) | 19.9  (4.5) | 18.3  (5.9) | 17.8  (5.2) |
| - Fascination (3 items) | 21.3  (6.1) | 21.9  (6.1) | 22.5  (5.4) | 21.6  (5.7) | 20.2  (6.6) | 20.0  (5.5) |
| - Being away (3 items) | 22.3  (5.9) | 23.6  (6.5) | 23.2  (6.0) | 21.4  (7.7) | 19.2  (8.2) | 18.4  (7.1) |
| - Coherence (3 items) | 20.3  (5.9) | 23.0  (6.0) | 22.4  (6.2) | 22.0  (4.6) | 20.2  (7.4) | 19.8  (5.8) |
| - Scope (2 items) | 15.3  (4.9) | 16.6  (3.8) | 16.5  (3.8) | 14.5  (4.5) | 13.7  (3.9) | 13.1  (4.3) |
